# Psychometric properties of a culture-adapted Spanish version of AIDA (Assessment of Identity Development in Adolescence) in Mexico

**DOI:** 10.1186/1753-2000-7-25

**Published:** 2013-07-31

**Authors:** Moises Kassin, Filipa De Castro, Ivan Arango, Kirstin Goth

**Affiliations:** 1 Mexican Institute of Transference Focused Psychotherapy, Iberoamerican University, Mexico City, Mexico; 2 Mexican Institute of Public Health Cuernavaca, México City, Mexico; 3 Borderline Personality Disorder Clinic, National Psychiatry Institute “Ramón de la Fuente Muñiz”, México City, Mexico; 4 Child and Adolescent Psychiatric Hospital, Psychiatric University Hospitals, Basel, Switzerland

**Keywords:** Identity, Questionnaire, Psychometrics, Adolescence, Cultural test adaption, Cross-cultural

## Abstract

**Background:**

The construct “identity” was discussed to be integrated as an important criterion for diagnosing personality disorders in *DSM-5.* According to Kernberg, identity diffusion is one of the relevant underlying structures in terms of personality organization for developing psychopathology, especially borderline personality disorder. Therefore, it would be important to differentiate healthy from pathological development already in adolescence. With the questionnaire termed *AIDA* (Assessment of Identity Development in Adolescence), a reliable and valid self-rating inventory was introduced by Goth, Foelsch, Schlueter-Mueller, & Schmeck (2012) to assess pathology-related identity development in healthy and disturbed adolescents. To test the usefulness of the questionnaire in Mexico, we contributed to the development of a culture-specific Spanish translation of *AIDA* and tested the reliability and aspects of validity of the questionnaire in a juvenile Mexican sample.

**Methods:**

An adapted Spanish translation of *AIDA* was developed by an expert panel from Chile, Mexico, and Spain in cooperation with the original authors, focusing on content equivalence and comprehensibility by considering specific idioms, life circumstances, and culture-specific aspects. The psychometric properties of the Spanish version were first tested in Mexico. Participants were 265 students from a state school (N = 110) and private school (N = 155), aged between 12 and 19 years (mean 14.15 years). Of these, 44.9% were boys and 55.1% were girls. Item characteristics were analyzed by several parameters, scale reliability by Cronbach’s Alpha, and systematic effects of gender, age, and socioeconomics by an analysis of variance (ANOVA). We evaluated aspects of criterion validity in a juvenile justice system sample (N = 41) of adolescent boys in conflict with the law who displayed various types of behavioral problems by comparing the *AIDA* scores of a subgroup with signs for borderline pathology (N = 14) with the scores obtained in the student sample using T-tests.

**Results:**

The psychometric properties of the Spanish version of *AIDA* proved satisfactory in the Mexican sample for items as well as scales. The reliability coefficients were α = .94 for the total scale “Identity Diffusion”, α = .85 and .92 for the two primary scales “Discontinuity” and “Incoherence”, and between α = .70 and .83 for the subscales. However, some items of the item pool in the Spanish version of *AIDA* did not meet all criteria for test equivalence and should thus be reformulated, taking the Mexican culture into account. Significant effects for gender and age were found. In line with our theory, the *AIDA* scores in the domains “Discontinuity” (high effect size) and “Incoherence” (medium effect size) were markedly higher in the delinquent boys than in the student group.

**Conclusion:**

The Spanish version of *AIDA* can be used in Mexico with satisfying psychometric properties, with only minor adaptions required. Our study contributes to the intercultural applicability of the *AIDA* instrument using the construct “identity integration vs. diffusion” as it was defined in the AIDA model for diagnostic purposes. Cultural differences, even those present in the various Spanish-speaking countries, should be modeled carefully.

## Background

The concept of “self” is regarded as an organizing construct in behavioral sciences, psychology, psychoanalysis, and social sciences [1]. However, the concepts of self, identity, and self-concept have been used indistinctly by various authors. Leary & Tangney [1] reported a list of 67 different terms that refer to constructs, processes, and phenomena related to “self”, “ego”, and “identity”. Moreover, the concept of “self” has been applied in various ways, e.g. as a synonym for the person as a whole, as a synonym for personality, as the subject of experience, as the system of beliefs about ourselves, and as one agent among others.

From a developmental point of view, typical phases and changes e.g. in the ego´s internal structures are described. These are thought to follow a sequential and predictable pattern over time, whereas each structure is regarded to improve the ability of attributing meaning to life´s experiences [2]. However, a broad range of different domains and constituents have been described in the literature which define the construct “identity” and related phases [3]. Moreover, individual development of domains may not occur in parallel. One domain might develop more readily or may be more organized than another. This results in highly individual identity patterns, probably associated with specific strengths, weaknesses, and even psychopathological traits [4]. Therefore, using broad concepts in studying identity with respect to developmental paths and possible changes over time seems to be adequate to promote scientific advancement.

Psychosocial and cultural influences are thought to play a major role in identity development throughout life [5,6]. In particular, the effects of the society on promoting (or hindering) development of the individual’s identity are of interest [7,8]. Moreover according to the narrative approach, an individual’s identity is shaped and modified by language and cultural aspects. Consequently, studies of identity should not only focus on absolute constructs but should take into account cultural factors, such as language, mentality, and living conditions.

Identity development is of prominent interest in the context of mental problems. In psychoanalytic and psychodynamic theories, the achievement of an integrated identity is regarded as central for healthy psychological development [9-11] and is viewed as a major task, especially in adolescence [12,13]. Severe disintegration is linked to the development of personality disorders, especially borderline pathology. In the operationalized psychodynamic diagnostic system (*OPD-2)*[14], problems related to identity are the central component of axis IV “structure”, extending from identity integration (structured-autonomous self) to disintegration (incoherent self). In the *DSM-IV*[15], identity disturbance (i.e. “markedly and persistently unstable self-image or sense of self,” p. 654) is included as one of the components of borderline personality disorder. For the new *DSM-5*[16,17], “identity” has been discussed extensively to be integrated as a key criterion for diagnosing personality disorders in general, in terms of reflecting one core impairment in self-related personality functioning in a dimensional way (see also Schmeck et al. in this issue).

We have previously described the different concepts of healthy and impaired identity development and presented a model combining psychodynamic, social-cognitive, and clinical psychology aspects [18] and providing an elaborated combination of the central subconstructs discussed in this field. This integrative model formed the basis of the self-report questionnaire *AIDA* (Assessment of Identity Development in Adolescence) to assess pathology-related identity development in adolescents aged between 12 to 18 years. The questionnaire, prepared by an international expert team, focused on conceptual clarity, the broad capturing of normal and impaired variants of expressing identity, ease of comprehension, and minimal confounding by factors such as culture, socioeconomics, age, and gender by developing appropriate (i.e. “fair”) item formulations (see below).

The items of *AIDA* are coded for pathology and add up to a total score reflecting the range extending from ”identity integration” to “identity diffusion“. To enable the identification of the scientific and historical rationale of the distinct subconstructs (e.g. compliance with goals, suggestibility, differentiated mental representations) and to promote research concerning possible specific relations to external variables or psychopathological subtypes, the subconstructs are formulated in terms of separated scales and subscales and are used as distinct units, although they are of course regarded as correlated and interacting in complex ways and to jointly form the higher-order phenotype “identity diffusion”.

**Figure 1:**
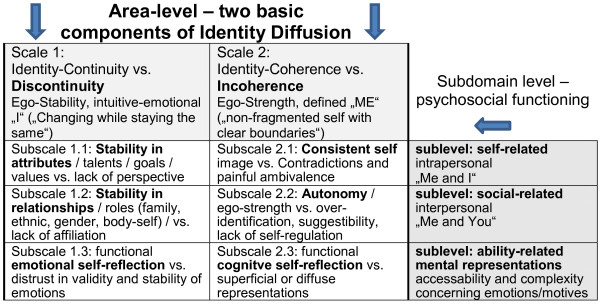
AIDA model for substructuring the construct “Identity Integration vs. Identity Diffusion” into theory-based areas (scales) and subdomains (subscales).

The distinction of the two main areas (primary scales), i.e. “Discontinuity” and “Incoherence”, is based on social-cognitive psychology (subjective vs. definitory self; see Figure 1) and the *OPD*-*2* definition of a healthy identity as leading to a “subjective feeling of continuity and coherence” [14]. The three subdomains reflect the central psychosocial or functional constituents used in several taxonomies, i.e. “self-related” vs. “social-related” vs. “ability-related”. This leads to a matrix consisting of six areas of pathology-related identity components.

*AIDA* showed good psychometric properties in a combined sample of German school children (N = 305) and patients of a Swiss clinic (N = 52) with excellent total score (Diffusion: α = .94), scale (Discontinuity: α = .86; Incoherence: α = .92), and subscale (α = .73-.86) reliabilities, justifying the use of theory-based subscales as distinct units [18,19]. An unrestricted exploratory factor analysis (EFA) on the item level showed a joint higher-order factor “identity integration” explaining 24.3% of variance, while the further 14 components did not match with reasonable units of shared content and only contributed minor explanatory power up to 62.6% in total. This was in line with the expected overall congruence on the phenotype level and was interpreted as an indicator for successful test construction, as all modeled contents/items had been constructed to reflect pathology-related identity development. However, the quality of a theory-based and pathology-oriented inventory, such as *AIDA*, hinges on the criterion validity, i.e. the potential to clearly differentiate healthy from impaired development. In a study in patients with personality disorder (N = 20) and healthy controls (N = 305), both areas of identity development, i.e. the two AIDA primary scales, demonstrated a remarkable discriminative power [18]. The “Discontinuity” scores differed between the groups with an effect size of d = 2.17 standard deviations and the “Incoherence” scores with d = 1.94 standard deviations (see also Jung et al. in this issue).

In Mexico, the mean age of the current population is 26 years. The population census 2010 revealed a serious problem with school drop-outs among Mexican adolescents and labeled 26% of adolescents aged between 15 and 19 years as “NINIs” (not studying, not working) [20]. As a consequence, adolescent delinquency has increased by 139% in the last six years. Adolescents not attending school or pursuing professional training are particularly at risk of getting involved with drug dealing or organized crime [21].

Mental disorders among adolescents in conflict with the law are common, with prevalences reported to be as high as 60-70% [22] or even 90% [23]. In a large US-American study [24] in 18,607 incarcerated adolescents, 70% of the boys and 81% of the girls showed severe psychiatric symptoms. In countries in which access to mental health services is limited, the prevalence of mental disorders in imprisoned subjects tends to be particularly high [22].

According to Kernberg [25,26] and Clarkin et al. [27], malignant narcissism and antisocial personality disorder are among the pathologies associated with borderline organization and are often seen in delinquent adolescents. Leichsenring, Kunst & Hoyer [28] found significant correlations between borderline personality organization (including identity diffusion) and antisocial personality disorder traits in violent offenders. Similarly, general behavioral and impulse control problems correlate with borderline personality organization [29].

Our goal was to investigate the relationship between identity development, delinquency, and development of personality disorders in Mexican adolescents. However, validated inventories with specific population norms for studying identity as well as specific pathologies in Mexico are lacking. Therefore, our Mexican team decided to contribute to the development of a Spanish version of the questionnaire *AIDA*, together with colleagues from Spain and Chile and in cooperation with the original authors. This instrument provides a broad conception, clear links to psychopathological traits, an established reliability and validity, and was constructed with a cross-cultural approach right from the start. Countries participating in the international AIDA study were requested to develop a culture-adapted AIDA version with equivalent content and satisfactory psychometric properties for all items and scales (see below) to enable international pooling of data and permit intercultural conclusions. In the current study, the culture-adapted Spanish translation of *AIDA* was tested in Mexican school children to evaluate the psychometric properties of items and scales of the questionnaire. The procedures used agreed well with those reported by the original authors for validating *AIDA* in Germany to provide clear comparability of the results. To evaluate aspects of criterion validity, we assessed a juvenile justice system sample (i.e. “conflict sample”) in which a higher frequency of psychopathological traits and identity diffusion was assumed. Content equivalence and successful test adaption were confirmed if the results obtained were similar those reported in the original study.

## Methods

### Participants and procedures

The three groups assessed consisted of students of a private school with assumed high socioeconomic background, students of a state school with assumed low socioeconomic background, and delinquent adolescents living in an institution. For the evaluation of the basic psychometric properties of the questionnaire *AIDA*, we pooled the students from the two schools to gain a heterogeneous sample. The ”conflict sample” was considered as a clinical subsample and was used to evaluate systematic differences in the levels of identity development. The study was approved by the local school authorities and the General Direction Treatment for Adolescents (DGTPA) in Mexico City. Ethical aspects were approved by the ethics committee of the Mexican Psychoanalytic Association.

#### School sample

The first group consisted of 155 subjects (66 boys, 89 girls) attending 1^st^-6^th^ grade of a private high school located in the Western area of Mexico City. Mean age was 14.8 years (SD 1.76) and ranged from 12 to 19 years, with only one student aged 19 years. The second group consisted of 110 students (53 boys, 57 girls) attending 1^st^-6^th^ grade of a state school located in the Northern metropolitan area of Mexico City. Mean age was 13.2 years (SD 1.30) and ranged from 12 to 18 years. Assessment of both student groups took place in the classroom during a 1-hour lesson. Prior to data collection, the students and parents signed a written informed consent that had been sent to them by the school administration one week before. About 50% of the required subjects participated in the study. The high missing rate was caused mostly by students not attending school on the assessment day. The participants were instructed to fill out the questionnaires alone, without discussing with their classmates. There was also the opportunity to clarify questions during completion of the questionnaire.

#### Conflict sample

The juvenile delinquents were recruited from a treatment center for adolescents in conflict with the law in the Southern area of Mexico City and consisted of 41 boys aged 15 to 18 years (mean age 16.4 years, SD 1.05), making up 55% of the center residents. Participants were those who had to stay at the institution during the 4-month test period (as part of a larger study, see below) and who were not sent to court on the assessment days. The education level of the adolescents varied between elementary to high school. The crimes committed by the adolescents covered the whole spectrum of delinquency, ranging from mobile phone theft to drug dealing, rape, and murder.

### Measures

The current study evaluating the basic psychometric properties of *AIDA* was part of a larger study to investigate the relationship between different aspects of identity development and mental health, using a number of self-rating questionnaires undergoing validation in Mexico. The results of this study will be reported elsewhere. Although not established yet, the Abbreviated Version of the Diagnostic Interview for Borderline (*Ab-DIB*) was used to obtain more specific information on the delinquent study group.

The *Ab-DIB*, published by Guile et al. [30], is a self-report screening measure for borderline psychopathology for adolescents. The *Ab-DIB* is derived from the *DIB-R* Interview (Revised Diagnostic Interview for Borderline) for adults developed by Zanarini et al. [31]. The questionnaire covers impulsiveness as well as affect-related and cognitive aspects of borderline personality (26 items). The *Ab-DIB* had previously been tested in Canadian suicidal youths (N = 139) for reliability and validity in comparison with *DIB-R* and Columbia Impairment Scale (*CIS*). Internal consistencies and test-retest intra-class correlations ranged from .80 to .86 and .77 to .95, respectively. Receiver operating characteristic (ROC) analysis yielded an area under the curve of .87 (p ≤0.001). Sensitivity was .88 and specificity ranged from .82 to .73, depending on the age range. Correlation of the *Ab-DIB*’s continuous scores with the *CIS* was .42 (p ≤0.001). Total scores of ≥12 (age 12-17 years) and ≥10 (age 18-21 years) were determined as the cut-off for borderline pathology. Validation of the Spanish translation of the *Ab-DIB* developed by our team is still in progress, and population norms or cut-off values for the Mexican population are not yet available. For an orientation, we used the Canadian cut-offs in an experimental fashion.

The original version of *AIDA* was developed in German and English at the same time by a Swiss-German-American research group. Special attention was directed towards culture-independent formulations and generic application of the constructs [19]. The resulting self-report questionnaire consisted of 58 items with a 5-step answering mode plus 6 semi-open questions for clinical use. In the study in German school children (N = 305) and patients from a Swiss clinic (N = 52), *AIDA* showed good psychometric properties with reliabilities α between .94 and .86 for the scales, and between .73 and .86 for the subscales (see Table 1) [18]. An EFA on the item level and the high scale intercorrelations confirmed a joint higher-order factor “identity integration”, supporting internal validity. Construct validity was also shown by the relationship with the external variable “maladaptive personality functioning“ assessed on the basis of the character scales of *JTCI 12-18 R* (Junior Temperament and Character Inventory; [32]). High levels of “Discontinuity” and “Incoherence” were associated with low levels in “Self-Directedness”, each regarded as an indicator of impaired self-related personality functioning. The criterion validity of the *AIDA* was high as shown by the clear discrimination between patients with personality disorder (N = 20) and healthy controls (i.e. remarkable effect sizes of d = 2.17 for the total score and between d = 1.04 and 2.56 standard deviations for the other scores). In both the original construction sample (N = 357 containing 1/6 psychiatric patients) [18] and the population sample (N = 1446 German students; Birkhölzer et al., in preparation), no systematic age effect on the AIDA scores was detected, suggesting that age-related normative levels of identity development do not exist as such, and that there is marked variability among adolescents. In contrast, a significant gender effect (approx. medium effect size), was found, with girls achieving higher AIDA scores, pointing towards more pronounced identity crisis or diffusion in girls than in boys.

**Table 1:** **Scale reliabilities** α **for AIDA in Germany**[18]**and for the Spanish version in Mexico (student sample N = 265) and marker items per subscale**

**Scales**	**α German**	**No. items**	**α Mexico**	**Marker items**
**Diffusion total score**	**.94**	**58**	**.94**	**Sum of scales**
**1. Discontinuity**	.86	27	.85	**Sum of subscales**
1.1 Attributes/ goals	.73	9	.70	1: I have hobbies or interests that are part of who I am; 33: As time goes by, I can imagine well how I will be in the future.
1.2 Relationships/ roles	.76	11	.74	2: I am proud of my roots and I feel like belonging to this group; 54: My friendships usually last only a few months
1.3 Emotional	.76	7	.76	3: I often don’t know how I feel right now.; 11: I'm not sure if my friends really like me.
self-reflection				
				
**2. Incoherence**	.92	31	.92	**Sum of subscales**
2.1 Consistency	.86	11	.83	4: I feel that I have different faces that do not fit together well. 13: I often feel lost, as if I had no clear inner self.
2.2 Autonomy	.84	12	.81	22: When my friends disagree with my opinion and ideas I feel "put down".; 42: When I’m alone I feel helpless.
2.3 Cognitive	.76	8	.75	51: I often have a block when I ask myself why I did things; 35: I am confused about what kind of person I really am.
self-reflection				

Translation of *AIDA* into different languages is in progress, under the supervision of the original authors. The process of translation and back-translation for the Spanish version of *AIDA* was done by an expert panel consisting of colleagues from Spain, Chile, and Mexico. For item translation, the main focus was on understanding the theoretical background of targeted constructs and achieving adequate translation or adaption of the items in a culture-specific fashion [33]. The items had to reflect the target content with words that were known and understood by the adolescents in that culture and that reflect the typical life circumstances. Moreover, the response patterns had to be similar, regardless of gender, age, socioeconomic status, ethnicity, or religiosity, avoiding classical “item bias”. For each item, medium probability “to say yes” had to be ensured [34]. For example, a German boy or girl would probably not state “I am proud of my roots”, even if it were true, while a Mexican boy or girl could state this without violating cultural norms. Thus, to reflect the subconstruct “identity-stabilizing cultural roles”, the item “I feel like belonging to my community” is a better choice for Germany to measure the same content using different words, respecting the differences in history, culture, and mentality. The first consensus version of the Spanish *AIDA* was approved by the original authors. For final approval, the psychometric properties have to be tested in each of the Spanish speaking countries. If necessary, different versions of the Spanish *AIDA* will have to be produced.

### Statistical analyses

To ensure cross-cultural comparability and enable international data pooling, translated AIDA versions should contain the same number of items per subconstruct with sufficient psychometric quality. Thus, a hierarchy of test procedures and statistical analyses is recommended [35].

Beta tests on small samples (e.g. 10-15 “balanced subjects” concerning health status, age, gender) are recommended as a first step to assess comprehensibility of the wording. Statistical analyses only refer to the number of missing values per item and typical response patterns, pointing to possible problems associated with inadequate wording. If there are more than 10% of missing values for any item or if the relevant question is answered mostly in the same way by the subjects, e.g. “completely yes” or “completely no” (i.e. excessively high or low percentage of symptomatic answers and therefore no discrimination between the individuals), this "problematic item" would have to be reconsidered and improved.

The recommended pilot test addresses the basic psychometric properties of the items and scales referring to the classical parameters of test validation but can be performed on a smaller sample that should be at least N = 24 for analyses on the subscale level and N = 116 for analyses on scale level (i.e. twice as many subjects as items per test unit). Statistical analyses refer to the number of missing values per item, percentage of symptomatic answers, age- or gender-related item bias, item total correlations, and resulting scale reliabilities Cronbach’s Alpha. If weak parameters for some items occur, these items would have to be reformulated and tested again until quality is satisfactory. The final validation sample should be highly representative for the target population and should integrate healthy subjects as well as subjects with psychopathological conditions to ensure a sufficiently large variance in the data and cover the full range of scales.

We used SPSS 19 for data analysis. In line with the validation procedure used for the original *AIDA*[18,19], we defined the following criteria: percentage of symptomatic answers p_i_ between 20-80% with an optimum of 50% and only single outliers of 5-95% per scale, effect size *f* of gender- or age-related item bias < .40, and item total correlation referring to the items’ scale and subscale r_it_ > .30. Scale reliabilities α were assumed to exceed .70 at scale level and .60 at subscale level, which is appropriate for heterogeneous contents, while homogeneity coefficients α > .80 would be very good and > .90 excellent.

To test for systematic differences in AIDA scores, a multivariate ANOVA was performed with the factors “gender” and “age”, descriptively divided into the age groups of early-to-middle (12–14 years) and middle-to-late (15–19 years) adolescence, in accordance with the procedure used for the original version of *AIDA*[18]. Additionally, we compared the results from the state school students and private school students to evaluate the impact of socioeconomic differences on identity development, controlled for age and gender effects. Score differences were evaluated not only for significance (1% level) but also for effect size *d*, conservatively calculated by (AM1-AM2) / ((SD1 + SD2)/2), and were assumed to reach at least a medium (>.50) but optimally a high (>.80) figure to avoid over-interpretation and artificial developmental differences.

Aspects of construct validity were evaluated by an EFA on item level (PCA with promax rotation) to take the assumed correlation between the contents into account and to optimize detectability of potential differences between the contents. Extraction criteria were eigenvalue >1 and the “elbow-criterion” in the scree plot for interpretation to highlight factors associated with eigenvalues above the slope in the curve. The procedure was similar to the one used in the original validation study to enable the comparison between phenotype dimensionality of *AIDA* in the Mexican sample and the factorial structure found in the German sample. Criterion validity was analyzed by T-test, comparing the AIDA results for the normal students with those of the group of delinquent adolescent boys displaying different types of behavioral problems. Additionally, we extracted a subsample from the delinquent sample by using their Ab-DIB scores and the Canadian cut-offs to gain a more homogeneous group with at least signs for borderline pathology.

## Results

### Item analysis and scale reliability

The beta test, performed for a group of 20 adolescents, ensured the basic comprehensibility of the items in the Spanish version of *AIDA* in the Mexican target population.

Statistical item analysis showed very good psychometric properties for the Spanish version of *AIDA* in Mexico. Most items showed only 0 to 2 missing values. This can be interpreted as a sign of good comprehensibility of the item wording and no apparent difficulties with respect to responding to the questions. Two items (items 44 and 46) were associated with 6 and 7 missing answers, respectively, but they represented less than 3% of the study population thus lying well below the 10% criterion.

All items matched the criteria for percentage of symptomatic answers (p_i_), reflecting how “easy” it is to answer an item in a symptomatic way, i.e. to say “yes” in our case (with all items coded towards identity diffusion before analysis). Mean percentage of symptomatic answers was 40%, and only 3 of the 58 items showed an extreme response pattern with a percentage below 10%. Thus, a good power of the items to truly discriminate between subjects with even very high or low characteristics in identity development can be assumed, as the full variety of the construct is covered by building the scales from “easy”, “medium”, and “difficult” items in total and therefore “ceiling” or “floor effects” are very unlikely.

Potential gender and age differences were analyzed by unidimensional ANOVAs to test for inherent item bias. This addressed the topic of “unfair items” which do not truly display differences but produce artificial differences by misleading wording. From the 58 AIDA items, only 10 showed significant differences between boys and girls that did not reach even a small effect size of f >0.10. Concerning the factor “age”, 9 items showed a significant intersubject effect that reached small effect sizes between 0.10 and 0.13 for 7 of them. Therefore, all items matched the preset criterion (effect size f < 0.40) and can be regarded as gender and age fair.

Most items fully matched the criteria for item total correlation (r_it_), reflecting the impact and weight of the item to constitute the assigned subscale or scale. Exceptions were items 8, 27, 12, and 20 that were below the preset criteria in one of the three categories: r_it_-coefficient in the assigned subscale and the assigned primary scale in the school sample, and, to give a special weight to the variance in the group of subjects with mental or behavioral problems, in the assigned subscale in the “conflict sample” (due to the small sample size, the analysis on primary scale level was not possible in the conflict sample). Thus, the four items were acceptable in general with only one problematic coefficient, but they could be improved by the wording of the question. For example, item 12 (“When people see me in new situations, they are very surprised how I can be.”), representing the content “observable contradiction” as part of the subscale “2.1 Incoherence-consistency”, showed an item total correlation r_it_ = .26 (below our criterion) in the subscale-referred analysis but r_it_ = .31 (above our criterion) in the scale-referred analysis and even r_it_ = .48 in the delinquent subsample. Three items showed weak r_it_ in more than one category (items 2, 33, and 49) and should be reconsidered to improve the assessability of the targeted construct in the Mexican population. A detailed description and suggestions for rewording are given in the discussion.

However, the higher-order category of psychometric property reflecting inner consistency “scale reliability Cronbach’s α” did not appear to be affected by the few weak items. Scale reliabilities were clearly above the preset criteria (see Table 1) in the pooled school sample.

### Distribution of the scales – effects of gender, age, and socioeconomics

Data for the total sample demonstrated a sufficient normal distribution of the scores with skewness and kurtosis displayed values around ׀1׀. The AIDA scores in the Mexican school sample differed with small to medium effect sizes (*d*) between the genders (see Table 2). The Mexican girls showed systematically lower AIDA scores than the boys. Moreover, systematic differences between the two age groups (i.e. 12-14 years and 15-19 years) were detected with small effect sizes (*d*) for Incoherence and medium effect sizes for Discontinuity. In a multivariate ANOVA with the full factor “age”, these differences only reached a small effect size (f = .13) for Discontinuity and no relevant effect size (f = .07) for Incoherence. Between the two school types, private school and state school, with assumed different socioeconomic backgrounds, no remarkable differences in the AIDA scores were detected after adjustment for gender and age. Although the group with higher socioeconomic status showed significantly lower scores (i.e. pointing to healthy integration) than the group from the state school (0.1% level) for all scales and subscales, the calculated effect sizes of these differences did only reach a relevant albeit small level for the Discontinuity score (f = .14).

**Table 2:** **Mean score (M) differences with associated effect sizes “*d” *concerning gender (girls N = 119, boys N = 146) and age group (12-14 N = 172, 15-18 N = 93)**

	**Gender differences**				**Age differences**			
	**Girls**	**Boys**			**12-14**	**15-19**		
	**M (SD)**	**M (SD)**	p*^1^	d*^2^	**M (SD)**	**M (SD)**	p*^1^	d*^2^
**Diffusion**	86.31	100.98	.000***	0.45	97.85	83,96	.001***	0.41
Total score	(36.00)	(29.66)			(31.26)	(37.04)		
1. Discontinuity	38.56 (16.77)	45.07 (14.58)	.001***	0.41	44.33 (14.06)	36.33 (18.29)	.000***	0.49
1.1 Attributes	14.56 (6.98)	16.32 (6.11)	.033*	0.27	16.03 (5.93)	14.13 (7.67)	.027*	0.28
1.2 Relationships	12.38 (7.90)	15.75 (7.49)	.000***	0.44	15.17 (7.39)	11.59 (8.17)	.000***	0.46
1.3 Emotional	11.62 (6.31)	12.99 (5.88)	.072	0.22	13.13 (5.78)	10.61 (6.50)	.001***	0.41
2. Incoherence	47.76 (22.99)	55.92 (18.92)	.002**	0.39	53.53 (21.07)	47.62 (22.15)	.034*	0.27
2.1 Consistency	15.20 (10.08)	19.20 (8.41)	.001***	0.43	17.57 (9.24)	15.97 (10.07)	.193	0.16
2.2 Autonomy	19.33 (9.21)	21.66 (7.57)	.028*	0.28	21.23 (8.38)	18.84 (8.74)	.030*	0.28
2.3 Cognitive	13.22 (6.71)	15.05 (6.20)	.024*	0.28	14.72 (6.38)	12.82 (6.69)	.024*	0.29

*^1^: Significance p * = 5%, ** = 1%, *** = 0.1% level.

*^2^: effect size d > 0.2 small, d > 0.5 medium, d > 0.8 big.

### Construct and criterion validity

**Figure 2:**
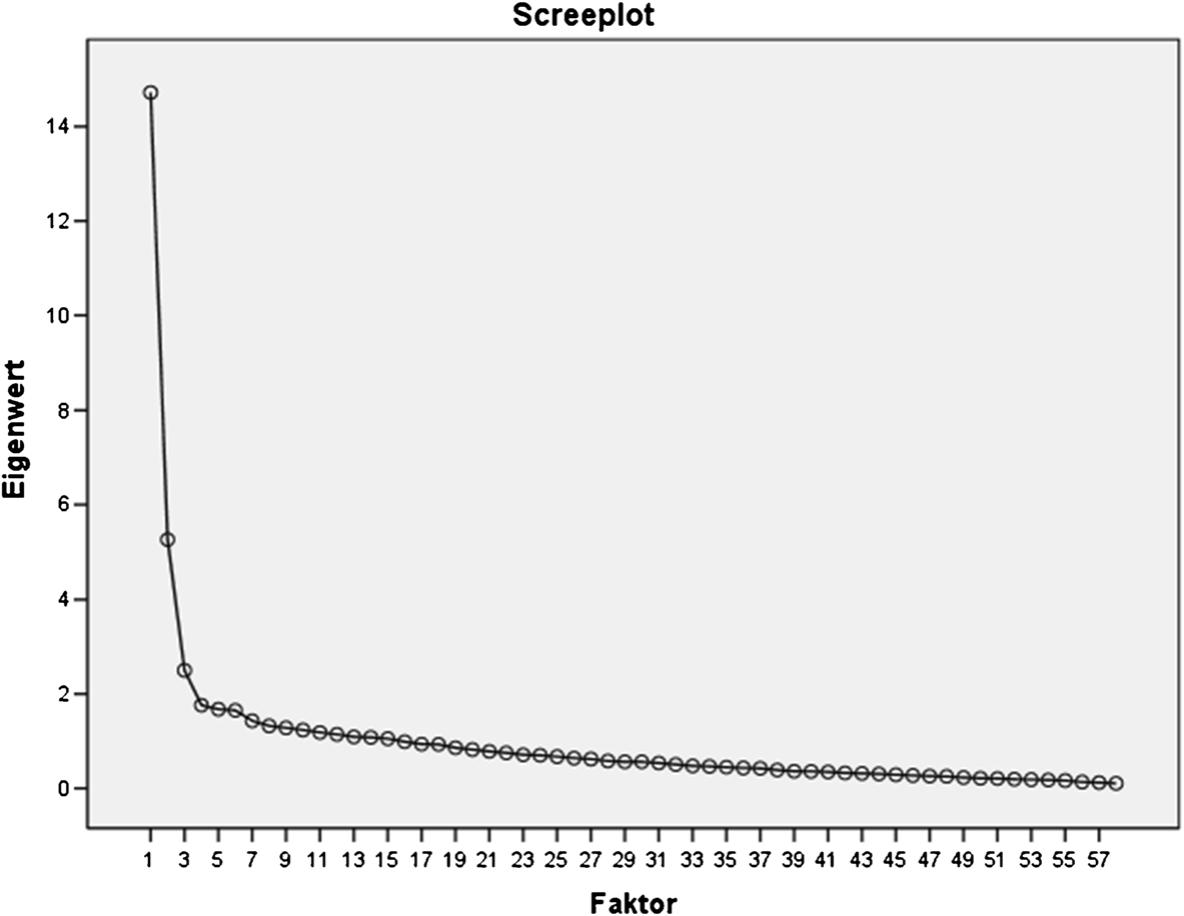
Scree prot for EFA on item level, extraced components explaining 66.2% of variance, first component 25, 4%.

The Spanish version of *AIDA* showed nearly the same factorial structure in the Mexican sample as in the German sample. In an unrestricted EFA, 15 components were detected that could not be interpreted reasonably in terms of phenotypically distinct subscales with shared content. The first component showed an eigenvalue of 14.7 accounting for 25.4% of the shared variance, and 43 of the 58 items showed their highest loading between .36 and .73 (mean .57) on this “i-factor”. A further 3 items contributed to the “i-factor” but with weak factor loadings of .28, .22, and .13. The second component above the “elbow-criterion” accounted for only 9.1% of the variance and combined 12 items from different subscales with no obviously shared content. The following components contributed only minor explanatory power (up to 66.2% in total; see Figure 2).

Except for subscales 2.1 and 2.2, the AIDA scores differed significantly in the expected direction (i.e. higher frequency of identity pathology in the conflict sample) between the students and delinquent adolescents. High effect sizes for the total score Diffusion (d = 0.93) and Discontinuity (d = 1.21) and medium effect size for Incoherence (d = 0.62) were obtained, while the subscales differed considerably in their impact. The Discontinuity subscales 1.1, 1.2, and 1.3 showed effect sizes of 0.90, 1.21, and 1.22, respectively. The Incoherence subdomains 2.1, 2.2, and 2.3 showed effect sizes of 0.55, 0.36, and 0.85, respectively. Fourteen of the delinquent adolescents showed signs of borderline pathology in the *Ab-DIB*. To additionally account for the age and gender effects, we compared a matching school sample to this “clinical” delinquent sample of boys over 14 years and found similar effect sizes (see Table 3).

**Table 3:** **Mean scores (M) and standard deviations (SD) of the Mexican school and delinquent sample (each subdivided) and associated effect size “*d”***

	**Mexican sample**					**Swiss-German sample**	
	**School sample boys+girls age 12-19**	**Delinquent sample only boys age > =15**	**School subsample only boys age > =15**	**Delinquent subsample +borderline pathology**	d *	**School sample N = 305**	**PD-patients N = 20**
	**N = 265**	**N = 41**	**N = 35**	**N = 14**			
	**M (SD)**	**M (SD)**	**M (SD)**	**M (SD)**		**M (SD)**	**M (SD)**
**Diffusion**	**92.92**	**119.93**	**94.80**	**125.21**	**0.84**	**65.87** (26.26)	**129.75** (32.57)
Total score	(34.01)	(35.84)	(32.79)	(40.08)			
1. Discontinuity	41.49	59.22	40.54	60.00	1.17	27.72 (11.49)	56.20 (14.74)
	(16.21)	(14.33)	(16.86)	(16.85)			
1.1 Attributes	15.35	21.73	15.43	21.21	0.79	12.95 (5.29)	20.75 (7.16)
	(6.65)	(5.68)	(7.10)	(7.58)			
1.2 Relationships	13.90	22.80	13.51	22.79	1.29	6.48 (4.78)	19.65 (6.82)
	(7.85)	(5.71)	(7.59)	(6.77)			
1.3 Emotional	12.24	14.68	11.60	16.00	0.68	8.30 (4.57)	15.80 (5.95)
	(6.15)	(5.82)	(6.56)	(6.40)			
2. Incoherence	51.43	60.71	54.26	65.21	0.46	38.15 (16.85)	73.55 (19.65)
	(21.60)	(23.58)	(19.93)	(25.88)			
2.1 consistency	17.00	21.20	19.17	21.21	0.22	12.65 (7.09)	30.95 (7.20)
	(9.56)	(8.30)	(9.75)	(9.12)			
2.2 Autonomy	20.38	22.54	21.60	24.00	0.26	15.21 (7.37)	24.30 (10.04)
	(8.57)	(9.75)	(7.01)	(11.53)			
2.3 Cognitive	14.05	16.98	13.49	20.00	0.94	10.29 (5.14)	18.30 (6.82)
	(6.54)	(7.42)	(6.37)	(7.52)			

*= differences between school subsample and delinquent subsample.

effect size d > 0.2 small, d > 0.5 medium, d > 0.8 big.

## Discussion

Assessment of identity development already in adolescence is important to study developmental paths in general and to enable valid conclusions about specific pathological risks. This is true especially in the light of the new revision of *DSM-5*, where “identity” has been discussed extensively to be integrated as a core criterion for impaired self-related personality functioning.

The Swiss-German-American questionnaire *AIDA* provides a reliable and valid assessment of the complex construct “pathology-related identity integration vs. identity diffusion” in adolescents by integrating different theoretical approaches and a reasonable structure of known subconstructs. Valid assessment tools must also meet the requirements of international usability in cross-cultural studies (e.g. as described by the International Test Commission; [35]), to model different phenotypes in different cultures and to enable valid comparisons of identity development in different societies by providing true equivalence in content of the assessment tool.

The Spanish culture-adapted translation of *AIDA* showed good psychometric properties in the Mexican sample, with similar patterns in results compared to the original version. We conclude that all constructs and subconstructs contained in the AIDA model to constitute “identity development” had been successfully transposed into the “Spanish-speaking culture” with good content equivalence.

However, detailed analysis revealed some problems on the item level in the Mexican sample. In the following, the results are discussed in detail, and suggestions for changes in item formulation with respect to the special need of the Mexican culture are presented. Each class of results is contrasted with the results of the original version to illustrate the special techniques and consequences of cultural test adaption.

Compared to the German items of *AIDA*, the Spanish items seemed to be “easier to answer in a symptomatic way”, i.e. to say “yes” coded towards identity diffusion in the Mexican school sample. While in the German sample, the mean percentage of symptomatic answers (p_i_) was 30% and 20 of the 58 items showed a percentage below 10% [19], in the Mexican sample the mean p_i_ was 40% and only 3 items showed an extreme value for “difficulty to be answered with yes” with a p_i_ below 10%. This means that the items were in general more difficult to answer with “yes” in the German version than in the Mexican version of *AIDA*. Thus, score differences between Mexican and German adolescents cannot be interpreted directly as different levels of identity diffusion because score equivalence cannot be assumed [33]. Therefore, population norms, e.g. T-values extracted from representative populations, have to be used for valid comparisons of samples concerning their “levels of identity development”.

All Spanish AIDA items matched the criterion for item bias and proved to be “age and gender fair” in the Mexican study. This agrees well with the results in the German study. The classical example for explaining “item fairness” is the “soccer item”: it would be unfair to judge the frequency of general physical activity by asking “How often do you play soccer” because girls usually do not like this game as much as boys do. Girls would probably say “never” more often, and therefore would be judged falsely as “physically inactive” in contrast to the boys. As all AIDA items can be regarded as gender and age fair, differences on the score level between gender and age groups can be interpreted as true developmental differences.

Not all Spanish items fully met the criterion for item total correlation (r_it_) in the Mexican sample. Four items (items 8, 27, 12, and 20) showed coefficients slightly below the criterion but would be generally acceptable for a translated version, especially taking into account that in all cases the item total correlations were excellent in the delinquent subsample with assumed behavioral problems. As the pooled school sample did not contain any subjects with diagnosed identity diffusion (e.g. a clinical sample with personality disorders), the data variance may not reflect the full range of true variability and relations in total, and the coefficients might be improved when “the pathological side” of the construct could better unfold its effects. Three items showed slightly weak r_it_ in more than one category or one coefficient far below the preset criterion (items 2, 33, 49). These items should be discussed in detail to detect a possible cultural bias in translation that might be eliminated by improved wording.

Item 2 (“I feel at home in my community, here is where I belong to”) showed the weakest r_it_ in the delinquent group, and we realized that in Mexico it might be difficult to feel ‘at home’ in a community suffering from a high crime rate. A better wording might be “I am proud of my roots and I feel like belonging to this group” to capture “Discontinuity-relations and roles” in terms of potential identity-giving and stabilizing cultural and/or ethnic roles.

Item 33 (“Just as I was as a child and as I am now, I can imagine how I could be in a few years”) was only slightly below the criterion and might be improved by a simpler wording, i.e. “As time goes by, I can imagine well how I will be in the future.”

Item 49 (“Many people are very "fake" and do not behave the way they really are”) showed a weak item total correlation with r_it_ = .11 in the subscale-referred analysis and r_it_ = .18 in the scale-referred analysis in the school sample, implying that this item has too little in common with the variance of the whole scale and the other items. Thus, it is not suitable for the scale “Incoherence-cognitive self-reflection” in terms of having shallow or superficial mental representations. At the same time, the item showed a high r_it_ = .55 in the delinquent subsample. This can be interpreted as a specific concordance with behavioral problems and may constitute improved quality of the assessment if psychiatric patients are included. Additionally, we realized that calling someone “a fake” is somehow “bad language” in the Mexican society and that students might refuse to respond to such unsuitable questions. To address this, the item should be expressed more politely, e.g. “Many people behave differently from what they really are” to adequately reflect the original wording of “not understanding complexity and variety of others’ behavior”.

Thus, for all “problematic” items, issues with cultural adaption of the contents were considered, and improved formulations were suggested. The high scale reliabilities α, with .94 for the total scale Identity-Diffusion, .85 and .92 for the two primary scales Discontinuity and Incoherence, and .70 to .83 for the subscales, are expected to further improve in the next pilot test with adapted item wording.

As in the German validation sample, the AIDA scores differed with about medium effect size between boys and girls in the Mexican school sample. However, in contrast to the findings in Germany, the Mexican girls showed systematically lower scores than the boys in the *AIDA*, implying healthier development, i.e. better identity integration. Therefore, differentiated norms for boys and girls should be extracted based on a representative Mexican population sample.

In contrast to the German subjects, Mexican subjects showed systematic differences between the two age groups (12-14 years and 15-19 years) with small to medium effect sizes. Therefore, it can be assumed that in Mexico distinct developmental stages related to age can be found. In line with the general theory of developmental identity, the younger adolescents displayed higher levels of “identity diffusion” without reaching pathological levels. This is viewed as a sign of an expected identity crisis at this age. Given this, differentiated age-specific population norms should be extracted in Mexico.

The socioeconomic background seemed to have no remarkable impact on the adolescents’ identity development in Mexico. Thus, students from different schools can be pooled for statistical analyses without affecting the results.

The EFA on the item level resulted in a very similar factorial structure as the one found in the Swiss-German validation sample. In the Mexican sample, 15 extracted factors explained 66.2% of the total variance with the first component alone explaining already 25.4%, while in the German sample, 15 extracted components explained 62.6% of the variance (first component 24.3%). This clearly documents favorable equivalence and effective culture-specific test adaption for assessing this complex construct in Mexico, as the translated version showed comparable patterns of results in a similar statistical analysis.

The confirmed “i-factor” is in line with the expected overall congruence on phenotype level, as all modeled contents (i.e. items) had been constructed to reflect current pathology-related identity development. The AIDA model combines distinct aspects of healthy identity concerning sources and/or consequences for its development and uses these theory-based distinct units in terms of scales and subscales experimentally to facilitate communication and research. However, empirical confirmation of the assumed structure is needed. As soon as different AIDA versions with convincing basic psychometric properties for item characteristics and scale reliability have been tested in normative samples, the instrument will be tested on the item and subscale level in a multi-sample study with a cross-cultural focus. Optimally, data obtained in various countries and continents should be suitable for pooling to analyze the underlying structure.

Because *AIDA* is a pathology-oriented inventory, the central quality standard lies in the diagnostic or predictive validity, i.e. the potential to differentiate healthy from impaired development. We evaluated criterion validity of the Spanish *AIDA* in Mexico by comparing the scores of the school sample to a “clinical sample” recruited from the juvenile justice system sample. The delinquent boys were a highly heterogeneous group with respect to their behavioral problems and comorbidities. The most probable behavioral problem in this subsample, i.e. “externalizing disorder”, is not assumed to be directly associated with severe identity diffusion in terms of “having no inner continuity and subjective self-sameness (Discontinuity)” or “having no consistently defined inner self-picture and autonomy (Incoherence)”. However, we clearly expected relevant consequences for identity development and detectable differences compared to the normal students as identity diffusion can be seen as a basis for several types of psychopathology, and the prevalence of mental disorders has proven to be high in incarcerated adolescents [22-24].

To create a more homogeneous contrast group, we used the Mexican pilot test version of the borderline screening inventory *Ab-DIB* with Canadian cut-offs. The subgroup of delinquent boys with signs of borderline pathology in this test (N = 14) was compared to the whole school sample on the one hand, and to a school subsample matched for age and gender on the other hand. In both analyses, the delinquent boys showed higher frequencies of identity pathology than the adolescents in the school sample, pointing towards satisfactory criterion validity of *AIDA* in Mexico. The Discontinuity scores differed with high effect sizes of d = 1.21 and 1.17, and the Incoherence scores with about medium effect sizes d = 0.62 and 0.46 standard deviations between the groups. However, the discriminative power of *AIDA* in this study was lower than in the original study that contained a true clinical sample of diagnosed PD patients. The strikingly different impact of the AIDA subscales on differentiating between the school and conflict samples implies that it is appropriate to treat the subscales as distinct units in an experimental fashion. Especially the subscales representing the psychosocial function “mental representation”, split into the domains “emotional” (part of Discontinuity; 1.3) and “cognitive” (part of Incoherence; 2.3), showed a different pattern compared to the other subscales. While subscale 1.3 showed a weaker discriminative potential than the other subscales of Discontinuity, 2.3 showed a stronger potential than the other subscales of Incoherence to differ between the school group and the “clinical” group. This may be due to the special characteristics of the delinquent sample with probable current behavioral problems like aggression and externalizing disorders that may be related to specific deficits leading to a special AIDA profile of this group.

### Limitations

A limitation of the study is the lack of psychiatric disorder assessment in the school sample. Based on epidemiological studies, we assumed that up to 15-20% of this representative sample of adolescents may exhibit minor to major signs of mental problems. However, without enrichment with a clinical subsample displaying extremer levels of identity diffusion, the heterogeneity of the sample for evaluating the basic psychometric properties of the Spanish *AIDA* is not optimal. Similarly, a true clinical sample of patients with defined diagnoses would be more informative for comparing their AIDA scores with those of the school sample in order to evaluate criterion validity. To extract population norms for Mexico, a representative sample with a higher participation rate is needed with adequate sample size in the different targeted groups for gender and age.

## Conclusion

The Spanish version of *AIDA* showed good psychometric properties in Mexico and can be used to assess the construct “pathology-related identity integration vs. diffusion” with reliability, validity, and content equivalence in comparison with the original AIDA questionnaire. This finding supports the cross-cultural generalizability of the underlying concept and confirms the importance of culture-specific test adaption in addition to literal translation of the questionnaire. Nevertheless, some items should be improved. Therefore, the test version of “*AIDA* Spanish – Mexico” should be further adapted and should be tested in a more heterogeneous population.

## Competing interests

The authors declare that they have no competing interests.

## Authors’ contributions

MK and KG were the main authors of the manuscript. MK and FC designed the study. KG performed the statistical analysis. IA wrote parts of the manuscript. MK collected the data. All authors read and approved the final manuscript.
